# Supplementary material for: Haplotype-Phased Chromosome-Level Genome Assembly of Cryptoporus qinlingensis, a Typical Traditional Chinese Medicine Fungus
Source: J Fungi (Basel). 2025 Feb 19;11(2):163. doi: 10.3390/jof11020163 (PMC11856377; doi:10.3390/jof11020163)
Supplement: Supplementary file 1 [file jof-11-00163-s001.zip › jof-3426466-supplementary.pdf]

Haplotype-phased chromosome-level genome assembly of *Cryptoporus qinlingensis*, a typical traditional Chinese medicine fungus

Yu Song[1], Ming Zhang[2], Yu-ying Liu[2], Minglei Li[3*], Xiuchao Xie[1*] and Jianzhao Qi[1,2,3*]

[1] Shaanxi Province Key Laboratory of Bio-resources, Qinba State Key Laboratory of Biological Resources and Ecological Environment (Incubation), School of Biological Science and Engineering, Shaanxi University of Technology, Hanzhong 723000, China.

[2] Shaanxi Key Laboratory of Natural Products & Chemical Biology, College of Chemistry & Pharmacy, Northwest A&F University, Yangling 712100, China.

[3] Center of Edible Fungi, Northwest A&F University, Yangling 712100, China

* Correspondence: mlli@nwafu.edu.cn (M.L.); xiexiuchao@126.com (X. X.); qjz@nwafu.edu.cn (J.Q.)

Content

Table S1. Statistics of PacBio Sequel II sequencing data mapping of *C. qinlingensis* SNUT genome.......1
Table S2. Statistics of Illumina NovaSeq sequencing data mapping of *C. qinlingensis* SNUT genome.......2
Table S3. Statistics of Hi-C sequencing data mapping of *C. qinlingensis* SNUT genome.......3
Table S4. Estimation of genome size of *C. qinlingensis* SNUT.......4
Table S5. Statistical table of *C. qinlingensis* SNUT assembly results.......5
Table S6. Statistical table of the length of the assembly sequence of *C. qinlingensis* SNUT.......6
Table S7. Statistics of BUSCO evaluation of *C. qinlingensis* SNUT genome.......7
Table S8. Genetic information the statistical table of protein-coding genes.......8
Table S9. Statistics of *C. qinlingensis* SNUT protein-coding gene annotation.......9
Table S10. Statistics of non-coding RNA annotation results in *C. qinlingensis* SNUT genome.......10
Table S11. The source (URL) statistics for 32 representative Basidiomycetes used to phylogenetic analysis......9
Table S12. Comparison of CAZyme-related gene families of 23 species.......10
Table S13. Statistics of *C. qinlingensis* SNUT repetitive sequence annotation results.......12
Table S14. Tandem repeat results statistics of *C. qinlingensis* SNUT.......13
Table S15. SSR analysis of *C. qinlingensis* and five macrofungi.......14
Table S16. Terpenoid biosynthesis related enzymes in *C. qinlingensis* SNUT genome.......16
Table S17. Core genes with multi-domain in *C. qinlingensis* SNUT genome.......18
Table S18. RIPP in *C. qinlingensis* SNUT genome.......19
Table S19. Identified and predicted PKS from macrofungi.......20
Figure S1. Kmer-Depth and Kmer Species-Frequency Distribution Plot.......22
Figure S2. Correlation analysis statistics between GC content and sequencing depth (Depth) of Hap-A.......23
Figure S3. GO, NR, SWISS, KEGG, COG Venn diagram of Hap-A.......24
Figure S4. GO, NR, SWISS, KEGG, COG Venn diagram of Hap-B.......24
Figure S5. Statistical Chart of KOG Functional Annotated Classification of Hap-A.......25
Figure S6. Statistical Chart of KOG Functional Annotated Classification of Hap-B.......25
Figure S7. KEGG Pathway Functional Classification Chart of Hap-A.......26
Figure S8. KEGG Pathway Functional Classification Chart of Hap-B.......26
Figure S9. Statistical map of functional annotation classification based on GO database of Hap-A.......27
Figure S10. Statistical map of functional annotation classification based on GO database of Hap-B.......27
Reference......28

Table S1. Statistics of PacBio Sequel II sequencing data mapping of *C. qinlingensis* SNUT genome.

Item	Value
Reads Number	5,545,070
Reads Bases (bp)	39,106,356,179
Largest Length (bp)	301,997
N50 Length (bp)	7,184
N90 Length (bp)	4,827
Average Length (bp)	7,052

Table S2. Statistics of Illumina NovaSeq sequencing data mapping of *C. qinlingensis* SNUT genome.

Item	Value
Insert size	450 bp
Raw data	6,433.3Mb
Clean data	6,369.3 Mb
Clean data Q20	97.12%
Clean data Q30	93.31%
Clean data GC	53.05%

Table S3. Statistics of Hi-C sequencing data mapping of *C. qinlingensis* SNUT genome.

Item	Value
Insert size	450 bp
Raw data	6,352.6 Mb
Clean data	6,213.2 Mb
Clean data Q20	97.85%
Clean data Q30	94.54%
Clean data GC	50.36%

Table S4. Estimation of genome size of *C. qinlingensis* SNUT.

Item	Number
kmer	21
genome_size	39.1M
Heterozygosity	0.21%
Repeat	21.2%

Genome size was estimated using genomescope v2.0 (http://genomescope.org/genomescope2.0/) software.

Table S5. Statistical table of *C. qinlingensis* SNUT assembly results.

Item	Value
Total Length	34,555,381
N50 length	2,645,904
N90 length	1,986,431
GC Content(%)	52.17%
N rate(%)	0

Table S6. Statistical table of the length of the assembly sequence of *C. qinlingensis* SNUT.

Chr	Length	Nrate	GC
Chr1	4000200	0%	52.15%
Chr2	3495755	0%	52.18%
Chr3	3321716	0%	52.37%
Chr4	2907634	0%	52.30%
Chr5	2671257	0%	52.41%
Chr6	2587211	0%	52.25%
Chr7	2483740	0%	51.82%
Chr8	2472269	0%	52.39%
Chr9	2400075	0%	52.28%
Chr10	2292593	0%	52.16%
Chr11	2198559	0%	52.51%
Chr12	1969942	0%	52.00%
Chr13	1489866	0%	52.47%
Chr14	87634	0%	25.82%

Table S7. Statistics of BUSCO evaluation of *C. qinlingensis* SNUT genome.

Item	Number	Percent (%)
Complete BUSCOs (C)	740	97.6
Complete and single-copy BUSCOs (S)	730	96.3
Complete and duplicated BUSCOs (D)	10	1.3
Fragmented BUSCOs (F)	3	0.4
Missing BUSCOs (M)	15	2.0
Total BUSCO groups searched (n)	758	100.0

Single-copy for single-copy BUSCOs; duplicated for multicopy BUSCOs; Fragmented for fragmented BUSCOs; Missing for missing BUSCOs. The predicted genes were assessed for completeness using the BUSCO software (version: 4.1.4) based on the fungi database (fungi_odb10).

Table S8. Genetic information the statistical table of protein-coding genes.

Item	Number	
	Hap-A	Hap-B
Genome size(bp)	34,555,381	34,378,451
Gene number	7,750	7,779
Gene total length(bp)	11,632,212	11,688,501
Gene average length(bp)	1,501	1,503
Gene length/Genome (%)	33.66	34.00

Table S9. Statistics of *C. qinlingensis* SNUT protein-coding gene annotation.

Sample	Item	Count	Percentage
Hap-A	All	7,740	100%
	Annotation	7,475	57.02%
	Swiss	4,413	53.61%
	Nr	7,475	57.02%
	GO	3,016	38.97%
	KEGG	3,117	40.27%
	COG	4,674	60.39%
Hap-B	All	7,768	100%
	Annotation	7505	96.61%
	Swiss	4413	56.81%
	Nr	7,505	96.61%
	GO	3,012	38.77%
	KEGG	3120	40.16%
	COG	4,672	60.14%

Annotation is the gene with at least one annotation; Uniprot is the gene annotated to the Uniprot database; Nr is the gene that is annotated to the Nr database; Interproscan is the gene that is annotated to the Interproscan GO is the gene annotated to the GO database; KEGG is the gene that is annotated to the KEGG database; Pathway is the gene that is annotated to the KEGG Pathway database; COG is the gene that is annotated to the COG database.

Table S10. Statistics of non-coding RNA annotation results in *C. qinlingensis* SNUT genome.

Sample	Class		number	totalLen(bp)	meanLen(bp)	% in Genome
Hap-B	tRNA		305	75	22,899	0.0663
	sRNA		0	0	0	0
	snRNA		16	142	2,273	0.0066
	rRNA_de	5S	5	114	570	0.0875
		5.8S	0	0	0	
		18S	6	1,898	11,388	
		28S	5	3,657	18,283	
	rRNA_ho	5S	-	-	-	-
		5.8S	-	-	-	
		18S	-	-	-	
		28S	-	-	-	
Hap-B	tRNA		296	75	22,273	0.0648
	sRNA		0	0	0	0
	snRNA		16	142	2,273	0.0066
	rRNA_de	5S	4	114	456	0.0721
		5.8S	0	0	0	
		18S	5	1,917	9,584	
		28S	4	3,687	14,749	

rRNA_ho	5S	-	-	-	-
	5.8S	-	-	-	
	18S	-	-	-	
	28S	-	-	-	

rRNA is ribosomal RNA; tRNA is transport RNA; sRNA is small regulatory RNA; snRNA is nucleolar small RNA. **totalLen** and **meanLen** are the total length and mean length.

Table S11. The source (URL) statistics for 32 representative Basidiomycetes used to phylogenetic analysis.

Species	Source
Ustilago maydis	https://www.ncbi.nlm.nih.gov/datasets/genome/GCF_000328475.2/
Marasmius oreades	https://www.ncbi.nlm.nih.gov/datasets/genome/GCF_018924745.1/
Lentinula edodes	https://www.ncbi.nlm.nih.gov/datasets/genome/GCF_021015755.1/
Physisporinus lineatus	https://www.ncbi.nlm.nih.gov/datasets/genome/GCA_027627245.1/
Abortiporus biennis	https://www.ncbi.nlm.nih.gov/datasets/genome/GCA_022606235.1/
Steccherinum ochraceum	https://www.ncbi.nlm.nih.gov/datasets/genome/GCA_004332605.1/
Panus rudis	https://www.ncbi.nlm.nih.gov/datasets/genome/GCA_022160315.1/
Cerrena zonata	https://www.ncbi.nlm.nih.gov/datasets/genome/GCA_038087015.1/
Phlebia brevispora	https://www.ncbi.nlm.nih.gov/datasets/genome/GCA_027627325.1/
Hermanssonia centrifuga	https://www.ncbi.nlm.nih.gov/datasets/genome/GCA_001913855.2/
Phlebiopsis gigantea	https://www.ncbi.nlm.nih.gov/datasets/genome/GCA_000832265.1/
Phanerochaete sordida	https://www.ncbi.nlm.nih.gov/datasets/genome/GCA_019973155.2/
Phanerochaete carnosa	https://www.ncbi.nlm.nih.gov/datasets/genome/GCF_000300595.1/
Trametopsis cervina	https://www.ncbi.nlm.nih.gov/datasets/genome/GCA_022385755.1/
Irpex lacteus	https://www.ncbi.nlm.nih.gov/datasets/genome/GCA_045517135.1/
Cytidiella melzeri	https://www.ncbi.nlm.nih.gov/datasets/genome/GCA_022592445.1/
Trametes gibbosa	https://www.ncbi.nlm.nih.gov/datasets/genome/GCA_022606055.1/
Polyporus arcularius	https://www.ncbi.nlm.nih.gov/datasets/genome/GCA_004369055.1/
Lentinus tigrinus	https://www.ncbi.nlm.nih.gov/datasets/genome/GCF_003813185.1/
Ganoderma sinense	https://www.ncbi.nlm.nih.gov/datasets/genome/GCA_002760635.1/
Dichomitus squalens	https://www.ncbi.nlm.nih.gov/datasets/genome/GCF_000275845.1/
Cryptoporus qinlingensis SNUT	This study

Obba rivulosa	https://www.ncbi.nlm.nih.gov/datasets/genome/GCA_001687445.1/
Gelatoporia subvermispora	https://www.ncbi.nlm.nih.gov/datasets/genome/GCA_000320605.2/
Sparassis crispa	https://www.ncbi.nlm.nih.gov/datasets/genome/GCF_003851025.1/
Amylocystis lapponica	https://www.ncbi.nlm.nih.gov/datasets/genome/GCA_022376435.1/
Antrodia cinnamomea	https://www.ncbi.nlm.nih.gov/datasets/genome/GCA_022598655.1/
Rhodofomes roseus	https://www.ncbi.nlm.nih.gov/datasets/genome/GCF_022264815.1/
Fomitopsis quercina	https://www.ncbi.nlm.nih.gov/datasets/genome/GCA_001632345.1/
Postia placenta	https://www.ncbi.nlm.nih.gov/datasets/genome/GCF_002117355.1/
Fibroporia radiculosa	https://www.ncbi.nlm.nih.gov/datasets/genome/GCF_000313525.1/

Table S12. Comparison of CAZyme-related gene families of 23 species.

species	GT	CBM	PL	CE	AA	GH
Phlebia centrifuga FBCC195	3	2	5	2	23	51
Ganoderma sinense ZZ0214-1	3	4	10	22	67	174
Ganoderma boninense G3	4	0	10	16	65	142
Ganoderma lucidum G.260125-1	4	2	9	18	58	146
Fomes fomentarius CIRM-BRFM1821	4	4	18	16	77	153
Trametes versicolor FP-101664	5	4	9	12	79	146
Cryptoporus qinlingensis SNUT	56	10	5	23	37	109
Dichomitus squalens LYAD-421	5	3	8	18	63	116
Cerioporus squamosus CCBS676	4	4	16	21	66	128
Ganoderma leucocontextum Dai12418	3	4	6	18	58	133
Trametes cingulata BRFM1805	2	8	9	13	64	135
Polyporus arcularius HHB13444	4	1	9	16	69	134
Phaner ochaetecarnosa HHB-10118-Sp	4	4	6	11	50	94
Irpex lacteus CCBASFr.238617/93	2	3	6	11	45	98
Polyporus squamosus CCBS676	3	3	13	12	45	85
Phaner ochaetechrysosporium RP-78	1	4	4	11	39	89
Ceriporiopsis subvermispora B	4	3	4	9	44	87
Trametes gibbosa CIRM-BRFM1770	6	3	10	9	39	117
Trametes maxima CIRM-BRFM1813	4	4	11	13	42	118

Trametes polyzona CIRM-BRFM1798	4	4	7	12	42	120
Irpex rosettiformis CBS384.51	4	5	5	8	49	111
Phlebia brevispora HHB-7030 SS6	3	15	6	10	44	112
Panus rudis PR-1116	4	12	11	11	51	106

Table S13. Statistics of *C. qinlingensis* SNUT repetitive sequence annotation results.

Type	number	totalLen(bp)	meanLen(bp)	In Genome (%)
LTR	3,336	5,705,104	1,738	16.5100
DNA	766	462,656	611	1.3389
LINE	462	227,089	498	0.6572
SINE	8	645	81	0.0019
RC	127	76,267	601	0.2207
scRNA	0	0	0	0
Unknown	6,966	2,843,800	434	8.2297
Total	11,665	9,206,229	823	26.6420

Table S14. Tandem repeat results statistics of *C. qinlingensis* SNUT.

Type	number	Repeat Size(bp)	Total Length(bp)	In Genome (%)
TRF	5,970	1~1,821	422,087	1.2215
Minisatellite DNA	4,233	10-60	225,739	0.6533
Microsatellite DNA	770	2-6	36,359	0.1052

Table S15. SSR analysis of *C. qinlingensis* and five macrofungi.

	Motif	No.	Percentage (%)	Length overall(bp)	Average Length(bp)	No. motifs type	Longest pattern
***Cryptoporus* sp. SNUT Hap A**	Monomer	48	4.12%	676	14.1	4	(T)$_{39}$
	Dimer	323	27.70%	4410	13.7	12	(AG)$_{13}$
	Trimer	574	49.23%	9735	17.0	59	(ATA)$_{14}$
	Tetramer	40	3.43%	888	22.2	32	(ATGG)$_{10}$
	Pentamer	40	3.43%	1265	31.6	38	(AGGAA)$_{30}$
	Hexamer	141	12.09%	8256	58.6	113	(GGGTTA)$_{35}$
	all SSRs	1166	100.00%	25230	21.6	258	(GGGTTA)$_{35}$
	**Motif**	**No.**	**Percentage (%)**	**Length overall(bp)**	**Average Length(bp)**	**No. motifs type**	**Longest pattern**
***Cryptoporus* sp. SNUT Hap B**	Monomer	49	4.20%	689	14.1	4	(T)$_{39}$
	Dimer	321	27.48%	4380	13.6	8	(AG)$_{13}$<br>(CA)$_{13}$
	Trimer	572	48.97%	9717	17.0	59	(ATA)$_{14}$
	Tetramer	41	3.51%	912	22.2	32	(ATGG)$_{10}$
	Pentamer	40	3.42%	1256	31.4	38	(AGGAA)$_{30}$
	Hexamer	145	12.41%	8382	57.8	116	(CCCTAA)$_{35}$<br>(TTAGGG)$_{35}$
	all SSRs	1168	100.00%	25336	21.7	257	(CCCTAA)$_{35}$<br>(TTAGGG)$_{35}$
	**Motif**	**No.**	**Percentage (%)**	**Length overall(bp)**	**Average Length(bp)**	**No. motifs type**	**Longest pattern**
Dichomitus squalens	Monomer	74	7.68%	957	12.9	4	(G)$_{18}$<br>(C)$_{18}$
	Dimer	286	29.67%	3902	13.6	8	(CG)$_{19}$
	Trimer	527	54.67%	10185	19.3	54	(GTC)$_{48}$
	Tetramer	21	2.18%	472	22.	18	(CAGC)$_{8}$
	Pentamer	10	1.04%	260	26.0	10	(ATGGG)$_{6}$<br>(GTGAT)$_{6}$
	Hexamer	46	4.77%	2256	49.0	43	(TAAAAT)$_{11}$
	all SSRs	964	100.00%	18032	18.7	137	(GTC)$_{48}$
	**Motif**	**No.**	**Percentage (%)**	**Length overall(bp)**	**Average Length(bp)**	**No. motifs type**	**Longest pattern**
Ganoderma sinense	Monomer	85	6.05%	1146	13.5	4	(T)$_{31}$
	Dimer	324	23.04%	4760	14.7	12	(GA)$_{50}$
	Trimer	790	56.19%	14391	18.2	58	(GTA)$_{32}$

	Tetramer	55	3.91%	1360	24.7	46	$(ATGG)_{19}$
	Pentamer	35	2.49%	1280	36.6	31	$(AGGGA)_{63}$
	Hexamer	117	8.32%	3696	31.6	109	$(GGGTTA)_{23}$
	all SSRs	1406	100.00%	26633	18.9	260	$(AGGGA)_{63}$
Lentinus tigrinus	**Motif**	**No.**	**Percentage (%)**	**Length overall**	**Average Length(bp)**	**No. motifs type**	**Longest pattern**
	Monomer	128	8.89%	2256	17.6	4	$(C)_{53}$
	Dimer	349	24.24%	5024	14.4	12	$(TC)_{26}$
	Trimer	825	57.29%	14139	17.1	51	$(ACA)_{22}$
	Tetramer	56	3.89%	1280	22.9	44	$(CAGG)_{19}$
	Pentamer	16	1.11%	480	30.0	15	$(ATCTT)_{11}$
	Hexamer	66	4.58%	2346	35.5	62	$(CCTAAC)_{15}$<br>$(ACCCTA)_{15}$<br>$(TTAGGG)_{15}$<br>$(CTAACC)_{15}$<br>$(CTAACC)_{15}$<br>$(TAGGGT)_{15}$
Polyporus arcularius	**Motif**	**No.**	**Percentage (%)**	**Length overall**	**Average Length(bp)**	**No. motifs type**	**Longest pattern**
	Monomer	135	8.56%	2227	16.5	4	$(C)_{59}$
	Dimer	269	17.06%	3594	13.4	12	$(AC)_{20}$
	Trimer	1055	66.90%	18540	17.6	50	$(ACC)_{19}$
	Tetramer	53	3.36%	1216	22.9	38	$(TAAG)_{9}$
	Pentamer	15	0.95%	420	28.0	15	$(TCTTG)_{8}$<br>$(AGATA)_{8}$
	Hexamer	50	3.17%	1854	37.1	45	$(GGGTTA)_{15}$
	all SSRs	1577	100.00%	27851	17.7	164	$(C)_{59}$
Trametes gibbosa	**Motif**	**No.**	**Percentage (%)**	**Length overall**	**Average Length (bp)**	**No. motifs type**	**Longest pattern**
	Monomer	699	21.63%	10259	14.7	4	(T)46
	Dimer	1060	32.81%	15298	14.4	8	$(GA)_{30}$
	Trimer	1224	37.88%	22278	18.2	57	$(GAT)_{31}$
	Tetramer	130	4.02%	3008	23.1	78	$(GAAA)_{17}$
	Pentamer	21	0.65%	640	30.5	20	$(CTCCT)_{17}$
	Hexamer	97	3.00%	4920	50.7	84	$(CTCCAT)_{48}$
	all SSRs	3231	100.00%	56403	17.5	251	$(CTCCAT)_{48}$

Table S16. Terpenoid biosynthesis related enzymes in *C. qinlingensis* SNUT genome.

Type	Entry	UniportKB database-based annotations	E-value	Indentify	Species	Accession Number
STS	YKJ-1000062.1	Delta(6)-protoilludene synthase STEHIDRAFT_25180	2e^{-130}	53.82%	*Stereum hirsutum* FP-91666 SS1	P9WEW0.1
	YKJ-1000063.1	Delta(6)-protoilludene synthase STEHIDRAFT_25180	1e^{-118}	47.92%	*Stereum hirsutum* FP-91666 SS1	P9WEW0.1
	YKJ-1007350.1	Sesquiterpene synthase 2	4e^{-153}	61.28%	*Postia placenta* Mad-698-R	A0A348B780.1
	YKJ-1001125.1	Sesquiterpene synthase Agr3	5e^{-172}	67.35%	*Cyclocybe aegerita*	A0A5Q0QU70.1
	YKJ-1001913.1	Longiborneol synthase CLM1	5e^{-26}	28.28%	*Fusarium graminearum* PH-1	I1S104.1
	YKJ-1001962.1	Terpene synthase 29	9e^{-33}	29.45%	*Postia placenta* Mad-698-R	A0A348B794.1
	YKJ-1001966.1	Alpha-cuprenene synthase COP6	3e^{-29}	30.77%	*Coprinopsis cinerea* okayama7#130	A8NCK5.1
	YKJ-1001968.1	Terpene synthase 29	8e^{-24}	27.03%	*Postia placenta* Mad-698-R	A0A348B794.1
	YKJ-1001973.1	Alpha-cuprenene synthase COP6	6e^{-31}	32.81%	*Coprinopsis cinerea* okayama7#130	A8NCK5.1
	YKJ-1002444.1	Alpha-cuprenene synthase COP6	3e^{-34}	33.33%	*Coprinopsis cinerea* okayama7#130	A8NCK5.1
	YKJ-1002889.1	Longiborneol synthase CLM1	5e^{-26}	28.28%	*Fusarium graminearum* PH-1	I1S104.1
	YKJ-1004240.1	Longiborneol synthase CLM1	7e^{-20}	27.73%	*Fusarium graminearum* PH-1	I1S104.1
	YKJ-1005356.1	Sesquiterpene synthase 10	3e^{-163}	64.07%	*Postia placenta* Mad-698-R	B8PD44.1

	YKJ-1005357.1	Sesquiterpene synthase 10	$1e^{-150}$	59.88%	*Postia placenta* Mad-698-R	B8PD44.1
	YKJ-1005439.1	Sesquiterpene synthase Agr1	$5e^{-120}$	50.46%	*Cyclocybe aegerita*	A0A5Q0QRJ3.1
	YKJ-1005443.1	Sesquiterpene synthase 3	$6e^{-157}$	64.51%	*Postia placenta* Mad-698-R	A0A348B781.1
	YKJ-1007120.1	Alpha-cuprenene synthase COP6	$2e^{-25}$	30.96%	*Coprinopsis cinerea* okayama7#130	A8NCK5.1
SQS	YKJ-1005860.1	Squalene synthase	0	81.29%	*Ganoderma lucidum*	A0SJQ5.1
PSY	YKJ-1004427.1	NADH dehydrogenase (ubiquinone) complex I, assembly factor 6	$2e^{-46}$	37.45%	*Homo sapiens*	Q330K2.2

Table S17. Core genes with multi-domain in *C. qinlingensis* SNUT genome.

Type	Entry	UniportKB database-based annotations	E-value	Indentify	Species	Accession Number
HCS	YKJ-1000252.1	Homocitrate synthase, mitochondrial	0	75.80%	*Schizosaccharomyces pombe* 972h-	Q9Y823.1
PKS	YKJ-1003626.1	Orsellinic acid synthase ArmB	0	41.92%	*Armillaria ostoyae*	A0A284RE13.1
NRPS	YKJ-1001472.1	Nonribosomal peptide synthase NPS2	0	46.69%	*Gelatoporia subvermispora* B	A0A248AFK6.1
	YKJ-1003785.1	L-2-aminoadipate reductase	0	51.72%	*Schizosaccharomyces pombe* 972h-	P40976.3
NRPS-like	YKJ-1003571.1	Adenylate-forming reductase Nps10	0	38.43%	*Heterobasidion annosum*	A0A1B1ZGB5.1
	YKJ-1003580.1	Adenylate-forming reductase Nps10	0	39.87%	*Heterobasidion annosum*	A0A1B1ZGB5.1
	YKJ-1004672.1	Adenylate-forming reductase Nps10	0	37.40%	*Heterobasidion annosum*	A0A1B1ZGB5.1
	YKJ-1004687.1	Adenylate-forming reductase Nps10	0	39.44%	*Heterobasidion annosum*	A0A1B1ZGB5.1
	YKJ-1004882.1	Adenylate-forming reductase Nps10	0	39.00%	*Heterobasidion annosum*	A0A1B1ZGB5.1
	YKJ-1001927.1	Oxalate--CoA ligase	$8e^{-57}$	32.41%	*Schizosaccharomyces pombe* 972h-	O74976.1

Table S18. RIPP in *C. qinlingensis* SNUT genome.

Entry	UniportKB database-based annotations	E-value	Indentify	Species	Accession Number
YKJ-1006151.1	UstYa family oxidase phomYb	$8e^{-13}$	28.40%	*Diaporthe leptostromiformis*	A0A142I729.1
YKJ-1006488.1	UstYa family oxidase phomYb	$7e^{-06}$	43.40%	*Diaporthe leptostromiformis*	A0A142I729.1
YKJ-1006512.1	UstYa family oxidase phomYd	0.001	28.95%	*Diaporthe leptostromiformis*	A0A8J9R8Y7.1
YKJ-1006514.1	UstYa family oxidase phomYe	$3e^{-09}$	26.77%	*Diaporthe leptostromiformis*	A0A142I739.1
YKJ-1007237.1	Oxidase ustYa	$1e^{-06}$	36.26%	*Aspergillus flavus* NRRL3357	B8NM67.1
YKJ-1007238.1	UstYa family oxidase phomYb	$1e^{-10}$	25.76%	*Diaporthe leptostromiformis*	A0A142I729.1
YKJ-1007267.1	UstYa family oxidase phomYb	$4e^{-10}$	25.76%	*Diaporthe leptostromiformis*	A0A142I729.1
YKJ-1007323.1	UstYa family oxidase phomYb	$1e^{-12}$	28.49%	*Diaporthe leptostromiformis*	A0A142I729.1

Table S19. Identified and predicted PKS from macrofungi.

Name	Source	accession number	Reaction substrates	Reference
ArmB	*Armillaria mellea*	I3ZNU9	orsellinic acid	[1]
PKS1	undescribed stereaceous basidiomycete BY1 *Agaricomycetes* sp	APH07629	orsellinic acid	[2]
PKS2	undescribed stereaceous basidiomycete BY1 *Agaricomycetes* sp	APH07628	orsellinic acid	[2]
PKS63787	*Antrodia cinnamomea*	KX683290	orsellinic acid	[3]
HerA	*Hericium erinaceus*		orsellinic acid	[4]
PKS15	*Moniliophthora roreri*	ESK96613	orsellinic acid	[5]

CC1G_05377	*Coprinopsis cinerea*	XP_001835415	orsellinic acid	[6]
CoPKS1	*Cortinarius odorifer*	OL512945	atrochrysone carboxylic acid	[7, 8]
CoPKS4	*Cortinarius odorifer*	OL512946	atrochrysone carboxylic acid	[7, 8]
CrPKS1	*Cortinarius rufoolivaceus*	OQ863313	atrochrysone carboxylic acid	[9]
CrPKS2	*Cortinarius rufoolivaceus*	OQ863314	atrochrysone carboxylic acid	[9]
CrPKS3	*Cortinarius rufoolivaceus*	OQ863315	atrochrysone carboxylic acid	[9]

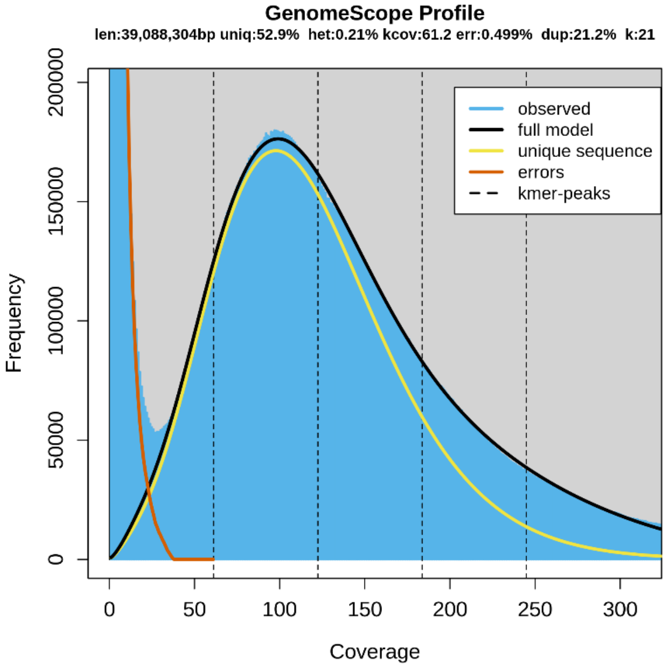

Figure S1. Kmer-Depth and Kmer Species-Frequency Distribution Plot.

The blue line represents the actual K-mer curve, the black line is the k-mer curve estimated by the model, the yellow line is the K-mer curve corresponding to the unique data, the red line represents the error curve due to sequencing errors, and the dashed line represents speculation K-mer peak.

Using the reads obtained by sequencing, K-mer-based analysis was used to estimate the genome size and heterozygosity. A K-mer refers to a sequence of K bp in length. Iteratively select a sequence of length K bases from a continuous sequence. If the length of the sequence is L and the length of the K-mer is K, then L-K+1 K-mers can be obtained. We take K-mers for the reads obtained by sequencing, and then count the frequency of each K-mer. According to the Lander waterman algorithm, the genome size (G) satisfies the following formula:

$$C_{base} = C_{k-mer} \times \frac{L}{L-K+1}$$

$$G = \frac{n_{k-mer}}{C_{k-mer}} = \frac{n_{base}}{C_{base}}$$

C base and C k-mer are the expected depth of coverage and K-mer, and n base and n k-mer are the total number of bases and the total number of K-mers in the sequence. In the case of a certain amount of data, the depth frequency of K-mer is subject to Poisson distribution, so the peak of the K-mer depth frequency distribution is the corresponding depth, which is used as an estimate of the expected depth of K-mer.

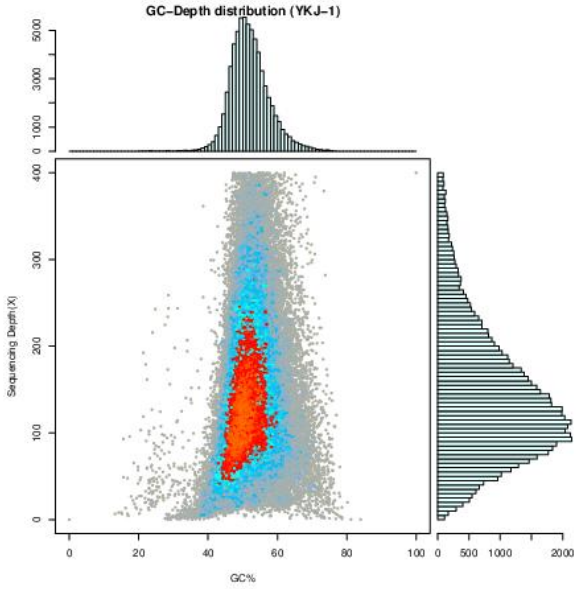

Figure S2. Correlation analysis statistics between GC content and sequencing depth (Depth) of Hap-A.

Horizontal coordinate indicates GC content, vertical coordinate indicates sequencing depth, right is the distribution of sequencing depth, and top is the distribution of GC content.

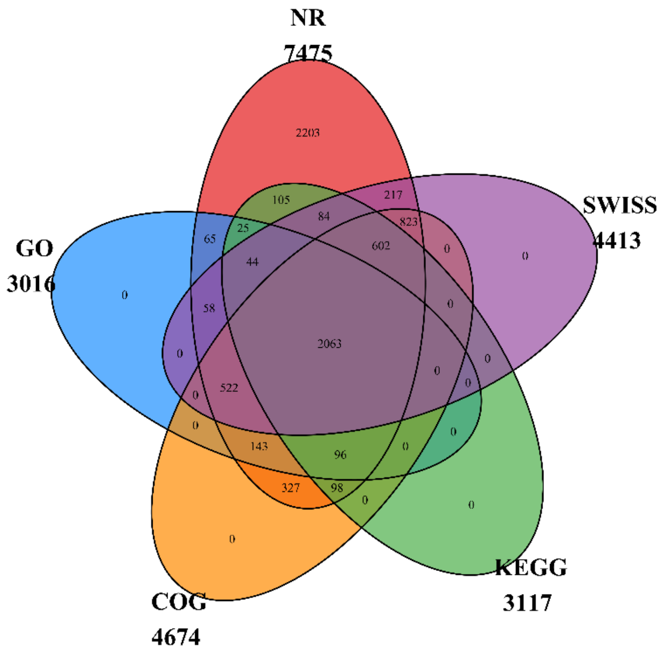

Figure S3. GO, NR, SWISS, KEGG, COG Venn diagram of Hap-A.

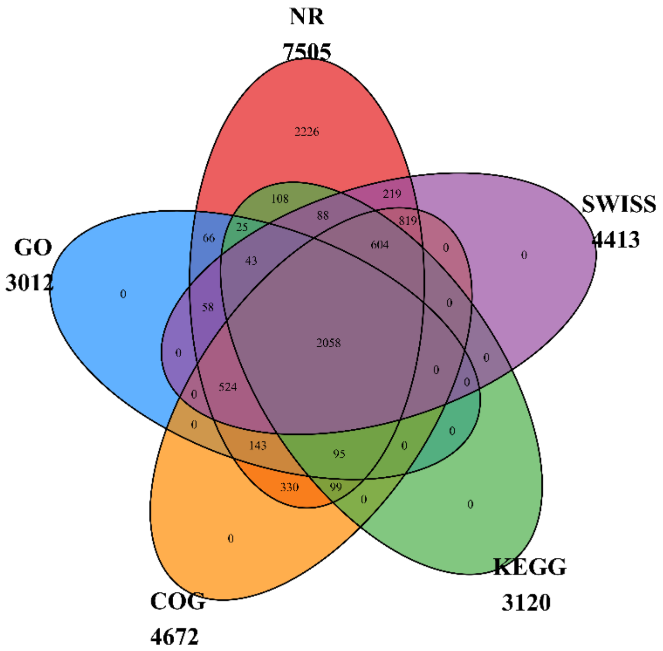

Figure S4. GO, NR, SWISS, KEGG, COG Venn diagram of Hap-B.

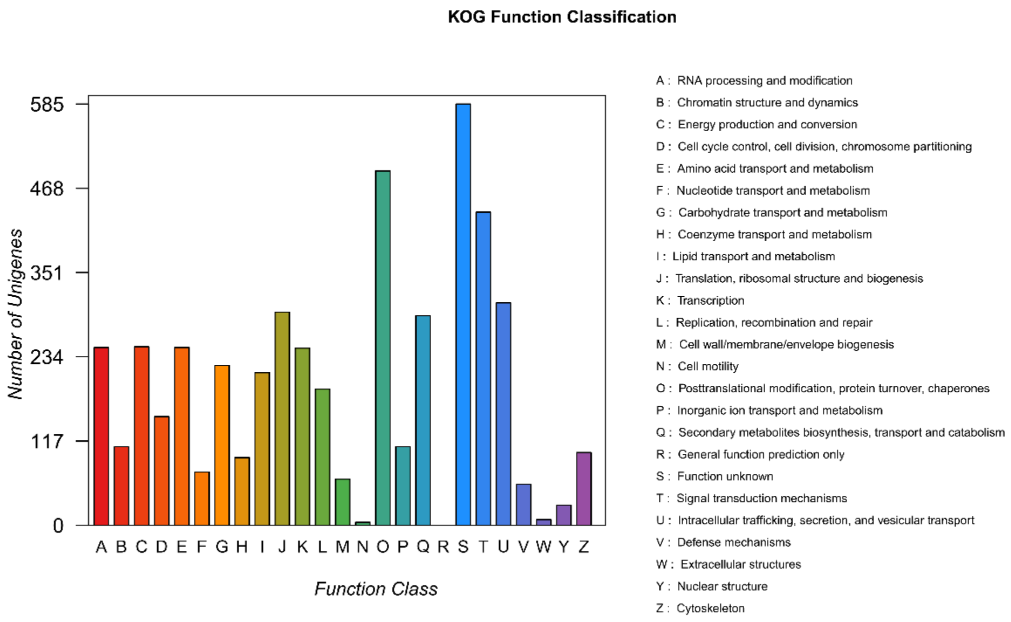

Figure S5. Statistical Chart of KOG Functional Annotated Classification of Hap-A.

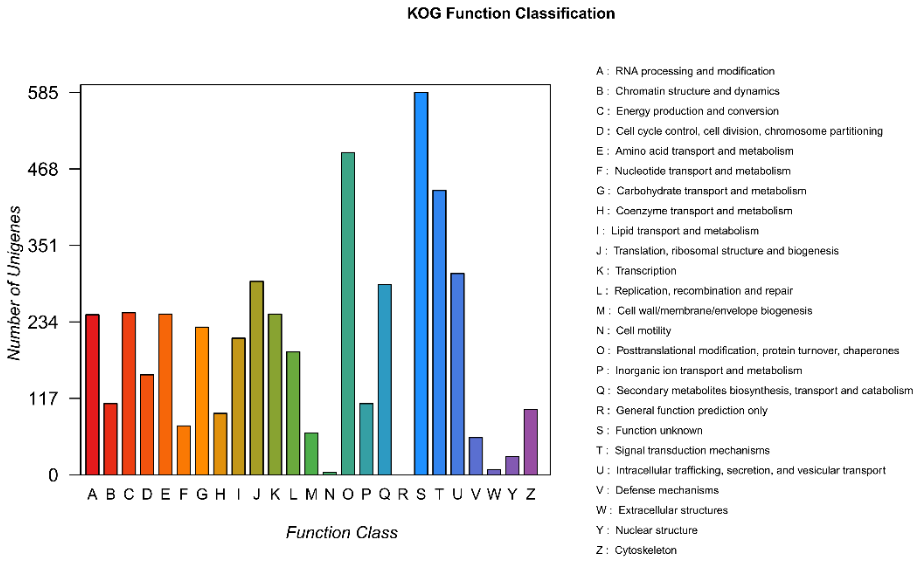

Figure S6. Statistical Chart of KOG Functional Annotated Classification of Hap-B.

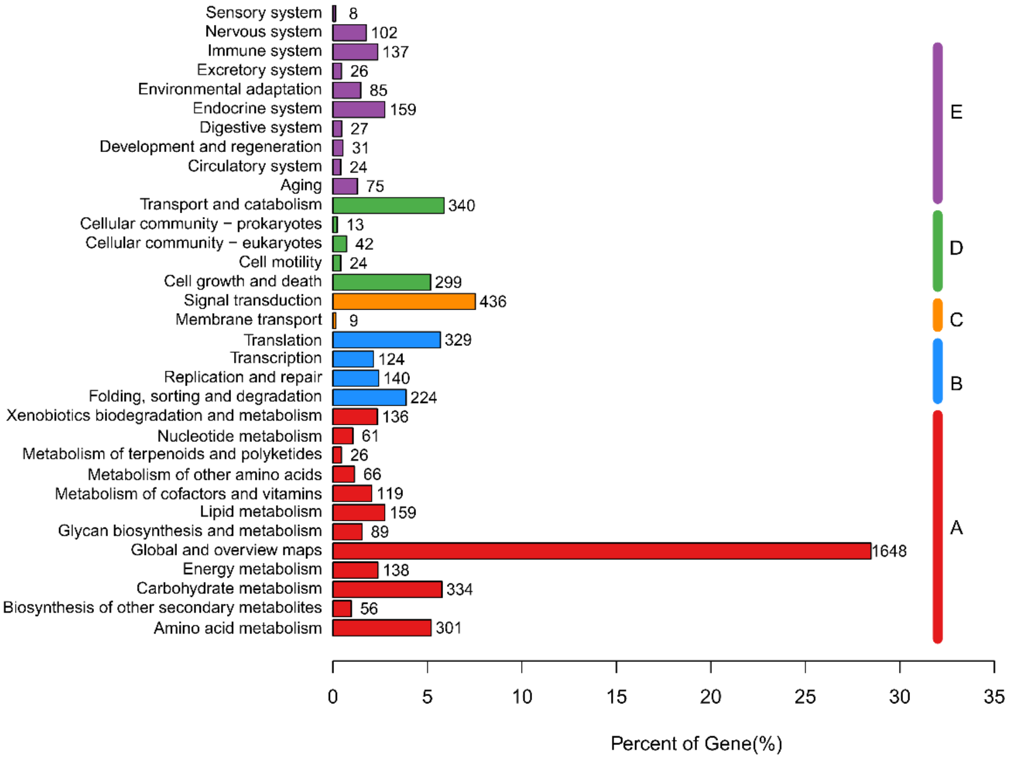

Figure S7. KEGG Pathway Functional Classification Chart of Hap-A.

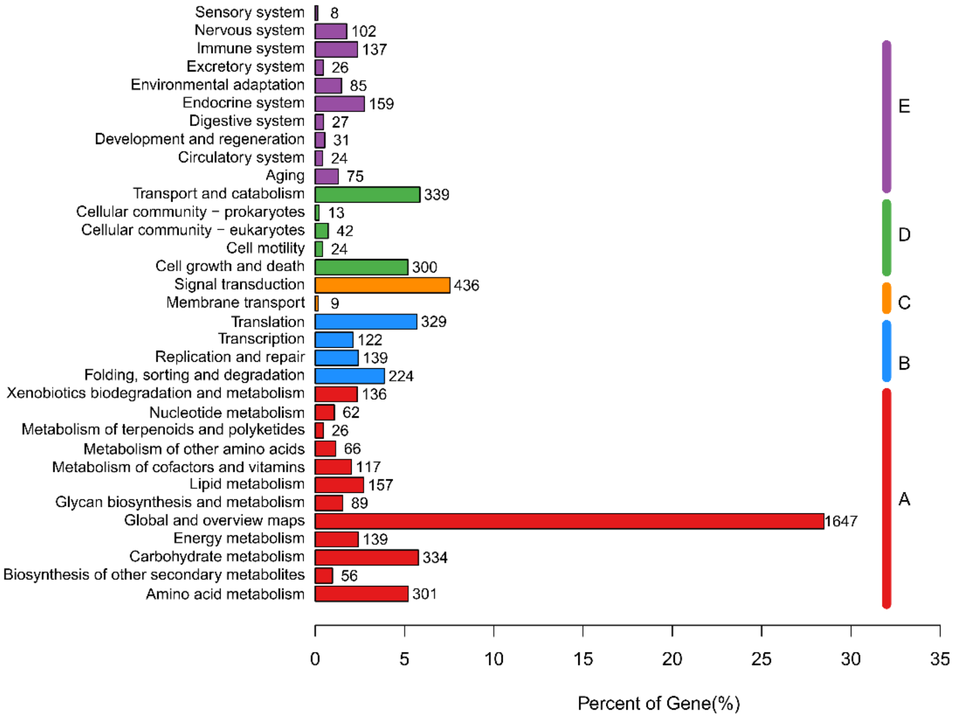

Figure S8. KEGG Pathway Functional Classification Chart of Hap-B.

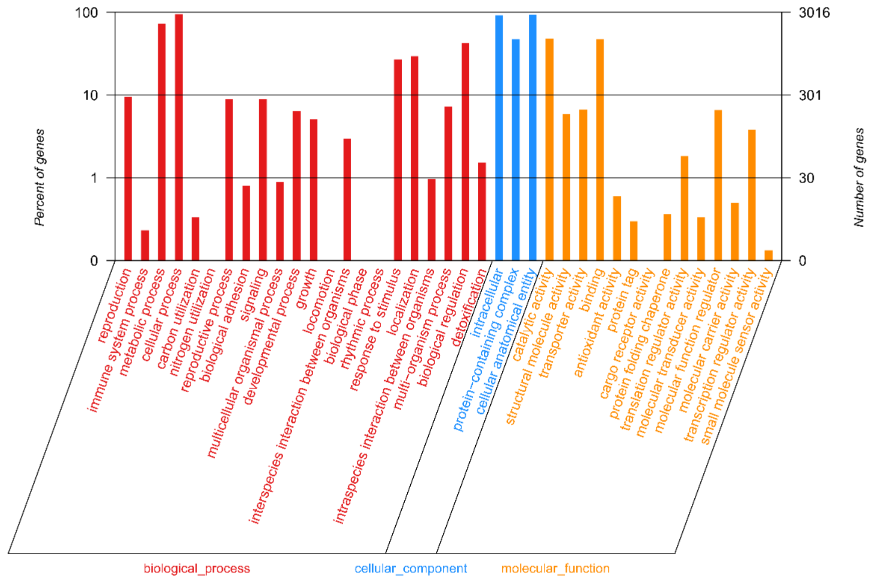

Figure S9. Statistical map of functional annotation classification based on GO database of Hap-A.

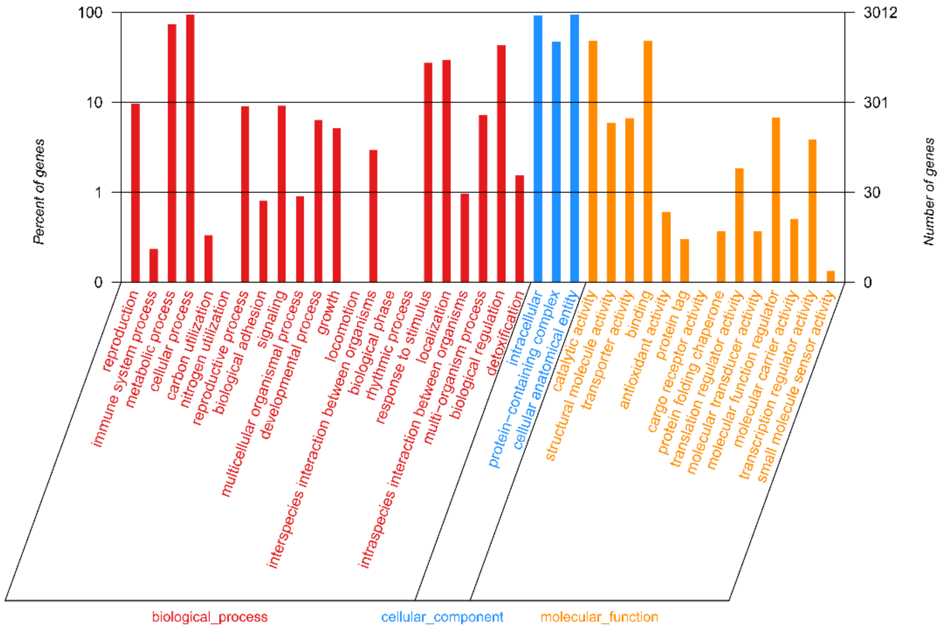

Figure S10. Statistical map of functional annotation classification based on GO database of Hap-B.

Reference

1. Lackner, G., et al., *Assembly of Melleolide Antibiotics Involves a Polyketide Synthase with Cross-Coupling Activity.* Chemistry & Biology, 2013. **20**(9): p. 1101-1106.
2. Braesel, J., et al., *Biochemical and genetic basis of orsellinic acid biosynthesis and prenylation in a stereaceous basidiomycete.* Fungal Genetics and Biology, 2017. **98**: p. 12-19.
3. Yu, P.W., et al., *pks63787, a polyketide synthase gene responsible for the biosynthesis of benzenoids in the medicinal mushroom Antrodia cinnamomea.* Planta Medica, 2016. **82**: p. 2.
4. Han, H., et al., *High-efficient production of mushroom polyketide compounds in a platform host Aspergillus oryzae.* Microb Cell Fact, 2023. **22**(1): p. 60.
5. Harvey, C.J.B., et al., *HEx: A heterologous expression platform for the discovery of fungal natural products.* Science Advances, 2018. **4**(4): p. 14.
6. Ishiuchi, K., et al., *Establishing a New Methodology for Genome Mining and Biosynthesis of Polyketides and Peptides through Yeast Molecular Genetics.* Chembiochem, 2012. **13**(6): p. 846-854.
7. Löhr, N.A., et al., *Unprecedented Mushroom Polyketide Synthases Produce the Universal Anthraquinone Precursor.* Angewandte Chemie-International Edition, 2022. **61**(24): p. 6.
8. Löhr, N.A., et al., *The Ketosynthase Domain Controls Chain Length in Mushroom Oligocyclic Polyketide Synthases.* Chembiochem, 2023. **24**(3): p. 7.
9. Lohr, N.A., et al., *Basidiomycete non-reducing polyketide synthases function independently of SAT domains.* Fungal biology and biotechnology, 2023. **10**(1): p. 17.
