# Supplementary material for: Haplotype-Phased Chromosome-Level Genome Assembly of Cryptoporus qinlingensis, a Typical Traditional Chinese Medicine Fungus
Source: J Fungi (Basel). 2025 Feb 19;11(2):163. doi: 10.3390/jof11020163 (PMC11856377; doi:10.3390/jof11020163)
Supplement: Supplementary file 1 [file Supporting File S1.pdf › Supporting File S1.pdf]

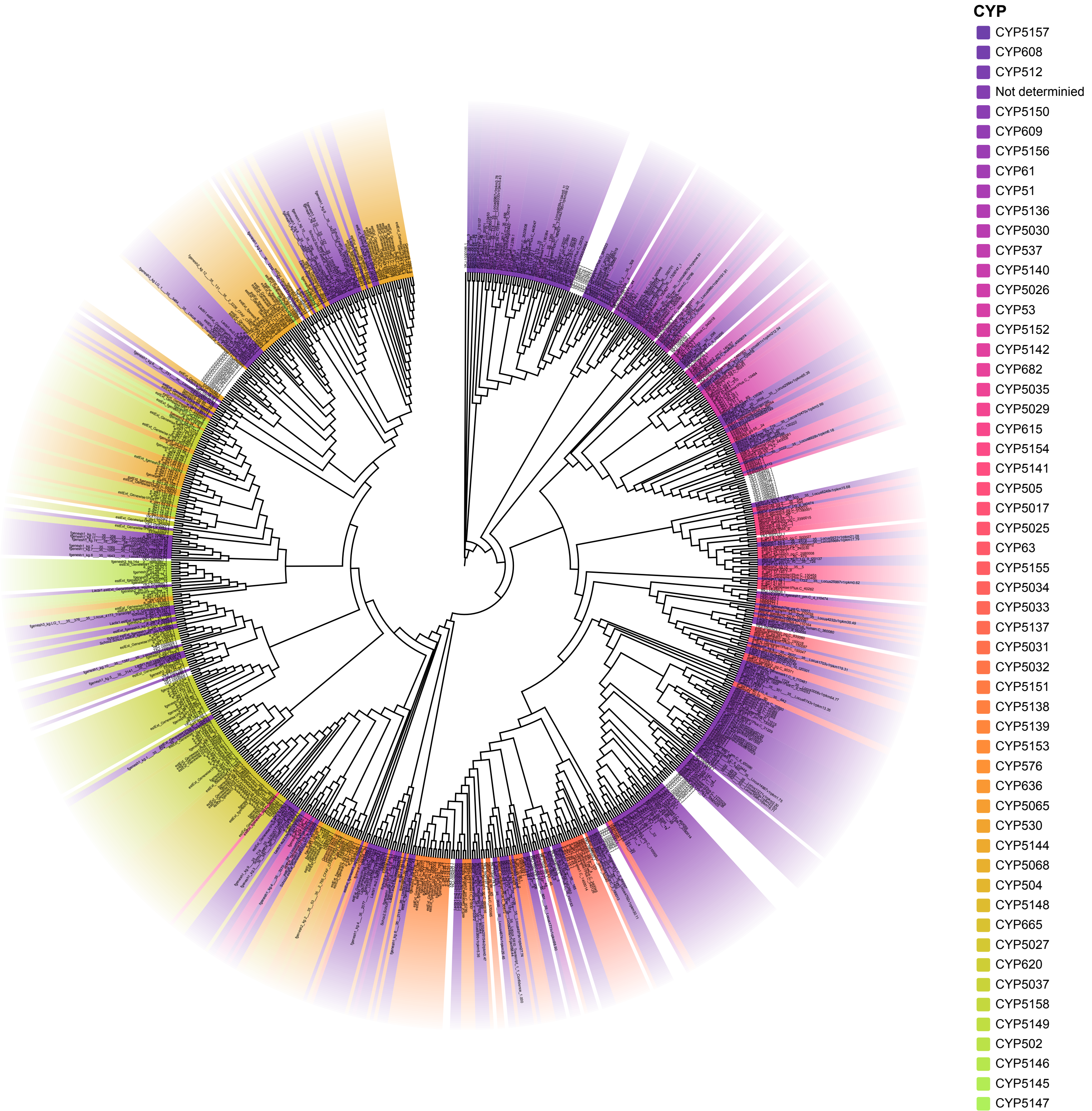
CYP
CYP5157
CYP608
CYP512
Not determinied
CYP5150
CYP609
CYP5156
CYP61
CYP51
CYP5136
CYP5030
CYP537
CYP5140
CYP5026
CYP53
CYP5152
CYP5142
CYP682
CYP5035
CYP5029
CYP615
CYP5154
CYP5141
CYP505
CYP5017
CYP5025
CYP63
CYP5155
CYP5034
CYP5033
CYP5137
CYP5031
CYP5032
CYP5151
CYP5138
CYP5139
CYP5153
CYP576
CYP636
CYP5065
CYP530
CYP5144
CYP5068
CYP504
CYP5148
CYP665
CYP5027
CYP620
CYP5037
CYP5158
CYP5149
CYP502
CYP5146
CYP5145
CYP5147
